# 

**Published:** 1907-05-15

**Authors:** 


					﻿Dental
Register
NELVILLE S. HOFF, Editor,
Ann Arbor, Mich.
Vol, LX1 --------MAY, 1907-------- No. 5
SAMUEL A. CROCKER & CO., Publishers
OHIO DENTAL AND SURGICAL DEPOT
35 W. FIFTH STREET, CINCINNATI, 0.
PRICE, ONE DOLLAR A YEAR
Entered at the Postoffice at Cincinnati, 0., as second-class mail matter.
				

